# Impact of Social Reward on the Evolution of the Cooperation Behavior in Complex Networks

**DOI:** 10.1038/srep41076

**Published:** 2017-01-23

**Authors:** Yu’e Wu, Shuhua Chang, Zhipeng Zhang, Zhenghong Deng

**Affiliations:** 1 Coordinated Innovation Center for Computable Modeling in Management Science, Tianjin University of Finance and Economics, Tianjin 300222, China; 2 School of Automation, Northwestern Polytechnical University, Xi’an 710072, China

## Abstract

Social reward, as a significant mechanism explaining the evolution of cooperation, has attracted great attention both theoretically and experimentally. In this paper, we study the evolution of cooperation by proposing a reward model in network population, where a third strategy, reward, as an independent yet particular type of cooperation is introduced in 2-person evolutionary games. Specifically, a new kind of role corresponding to reward strategy, reward agents, is defined, which is aimed at increasing the income of cooperators by applying to them a social reward. Results from numerical simulations show that consideration of social reward greatly promotes the evolution of cooperation, which is confirmed for different network topologies and two evolutionary games. Moreover, we explore the microscopic mechanisms for the promotion of cooperation in the three-strategy model. As expected, the reward agents play a vital role in the formation of cooperative clusters, thus resisting the aggression of defectors. Our research might provide valuable insights into further exploring the nature of cooperation in the real world.

Although competition and natural selection among species drive their evolution and theoretically bring more benefit to defection, the emergence of cooperation behaviors among agents is still ubiquitous in the real world, ranging from biological systems to economic and social systems^1^,^2^,^3^,^4^,^5^. Therefore, exploring the extensive cooperation behaviors becomes an open challenge and attract the attentions of scientific researchers in a myriad of fields including physics, mathematics, biology and behavioral science^6^,^7^,^8^,^9^,^10^. In addition to being a good tool for the decision problem^11^,^12^,^13^,^14^, evolutionary game theory has been generally accepted as the common framework to tackle the issue. In particular, the prisoner’s dilemma game (PDG), the snowdrift game (SDG) and the stag-hunt game (SHG) are often employed as the metaphors for the social dilemmas^15^,^16^,^17^,^18^,^19^,^20^,^21^.

Irrespective of which game applies, agents can choose either to cooperate or to defect in the procedure of the game. They will both receive *R (P*) under mutual cooperation (mutual defection).A defector will obtain the temptation *T* when confronts with a cooperator, while the cooperator gains the so-called sucker’s payoff *S*. As a standard practice, in the PDG, the payoffs must be ordered as *T* > *R* > *P* > *S* so that the defection is the best strategy irrespective of the opponent’s decision^22^,^23^,^24^,^25^. Both in the SDG and the SHG, players interact in a similar way, but the payoff ranking is *T* > *R* > *S* > *P (R* > *T* > *P* > *S*) for the SDG (SHG). This minor distinction induces a significant change in the game dynamics^26^.

Over the past decades, a variety of scenarios have been proposed to offset the above unfavorable outcome and to enhance the cooperation in the population. These mechanisms can be classified into five categories: kin selection, direct and indirect reciprocity, group selection and network reciprocity^7^. In particular, network reciprocity, where agents are arranged on the spatially structured topology and interact only with their direct neighbors, has attracted a great deal of attention^23^,^27^,^28^. Therefore, the evolution of cooperation has been extensively explored in a variety of topologies such as regular square lattices, small-world networks, Erdös-Rényi (ER) graph, Barabási-Albert scale-free (SF) network and so on^29^,^30^,^31^,^32^. Meanwhile, different factors have also been considered in the same network structures for exploring their impact on the evolution of cooperation. For instance, different evolutionary dynamics^33^, different rewire mechanisms^34^, high value of the clustering coefficient^35^, randomness of different nature or a large set of strategies^36^, all allow the emergence and prosperity of cooperation, even if the temptation to defect reaches a high value^37^,^38^,^39^.

Up to the present, many realistic scenarios are introduced into evolutionary games such as tit-for-tat, win-stay and lose-shift, memory effects, age structure, and different teaching capabilities^40^,^41^,^42^,^43^,^44^. Besides, there is another situation of particular relevance that has received a lot of attention^45^,^46^,^47^,^48^,^49^,^50^. This is the case of social reward where cooperators can get additional benefits for their selfless behavior. In most of existing works under the reward mechanism, cooperators receive rewards as a second-stage behavior^51^, and these papers are almost limited to pubic goods games^52^,^53^,^54^. Inspired by these findings, we wonder how cooperation fares when reward is considered as an independent strategy in the pairwise interaction games.

In this work, we explore the impact of social reward on the evolution of cooperation on 2-person games in which interactions are driven by complex topologies. Specially, we introduce a third strategy (reward) in the traditional PDG and SDG and study how these defined ‘reward agents’ affect the evolution of cooperation in several topological settings.

## Results

A third strategy, reward, as an independent yet particular kind of cooperation strategy is introduced in the traditional PDG and SDG models to explore the influence of social reward on the emergence of cooperative behavior. When a reward agent encounters a cooperator, the former will reward the latter *γ* (the degree of reward) at a small cost *β* with the effect of increasing the effective payoff gained by the latter. The simulation results (see Supplementary Fig. S1 and Fig. S2) show that only when the value of *β* is smaller than *γ*, will the reward mechanism promote the evolution of cooperation. When *γ* is fixed, the smaller the value of *β*, the more obvious the promoting effect. In order to get an obvious enhancement, we fix *β* = 0.01 and change *γ* from 0.1 to 0.9 in the whole work. The details of interactions between agents and their corresponding payoffs are summarized in Table 1 in the *methods* section.

**Figure 1:**
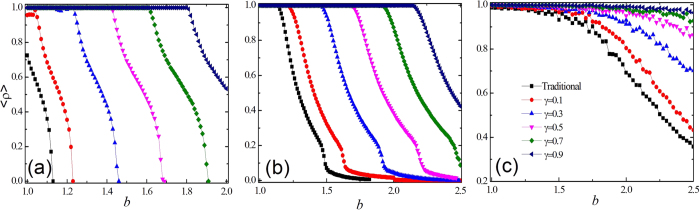
Fraction of cooperative agents (cooperators and reward agents) <*ρ*> as a function of *b* for different values of *γ* and for the considered three types of network in the prisoner’s dilemma. (**a**) Square lattice, (**b**) ER graph and (**c**) SF network. All the results in Fig. 1 have been obtained for *N* = 10^4^ nodes, <*k*> = 4, and *β* = 0.01.

Firstly, we concentrate on the impact of the introduced mechanism on the sustainability of cooperation by measuring the average fraction of cooperative agents <*ρ*> which is defined as the total fraction of cooperators and reward agents presented at the equilibrium state. The results for the PDG on three types of networks (see Methods) are shown in Fig. 1. In the standard settings of the games, the PDG on a regular square lattice provides smaller levels of cooperation compared with the other two networks ? Thus, the case of the PDG on a regular square lattice is firstly considered as demonstrated in Fig. 1(a). For the traditional PDG (i.e., no social reward), the fraction of cooperators decreases rapidly and becomes zero soon afterwards for a very small value of temptation b as indicated in the black dot line in Fig. 1(a). Interestingly, even if a small reward (*γ* = 0.1 or 0.3) is introduced, the dynamics of the system radically change: cooperation can survive and even become the dominant strategy for some values of *b*. The simulation results for the ER and the SF networks are presented in Fig. 1(b) and Fig. 1(c), respectively. It is observed that the larger the *γ* value, the higher the critical value of *b* for the extinction of cooperators, which indicates that the reward mechanism facilitates the evolution of cooperation under the two networks as well.

**Figure 2:**
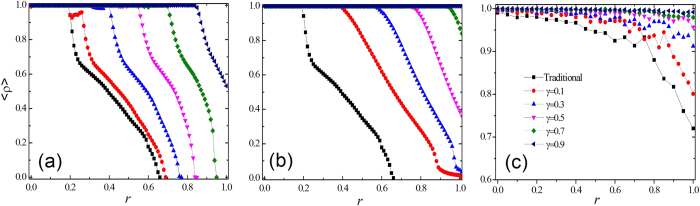
Fraction of cooperative agents (cooperators and reward agents) <*ρ*> in dependence on the cost-to-benefit ratio *r* for different values of *γ* and for the considered three types of network in the snowdrift game. (**a**) Square lattice, (**b**) ER graph and (**c**) SF network. Other parameters are consistent with Fig. 1.

The generality of this newly introduced mechanism in promoting cooperation is also tested for the SDG. The fraction of cooperative agents <*ρ*> as a function of the cost-to-benefit ratio *r* for the three topologies is presented in Fig. 2. It is observed that cooperation can survive for a wider range of *r* values for a larger *γ*, implying that the reward mechanism boosts the evolution of cooperation. The results of numerical simulation in the PDG and the SDG suggest that the social reward mechanism is generally effective in sustaining the evolution of cooperation, irrespective of potential interaction networks and the types of evolutionary games.

**Figure 3:**
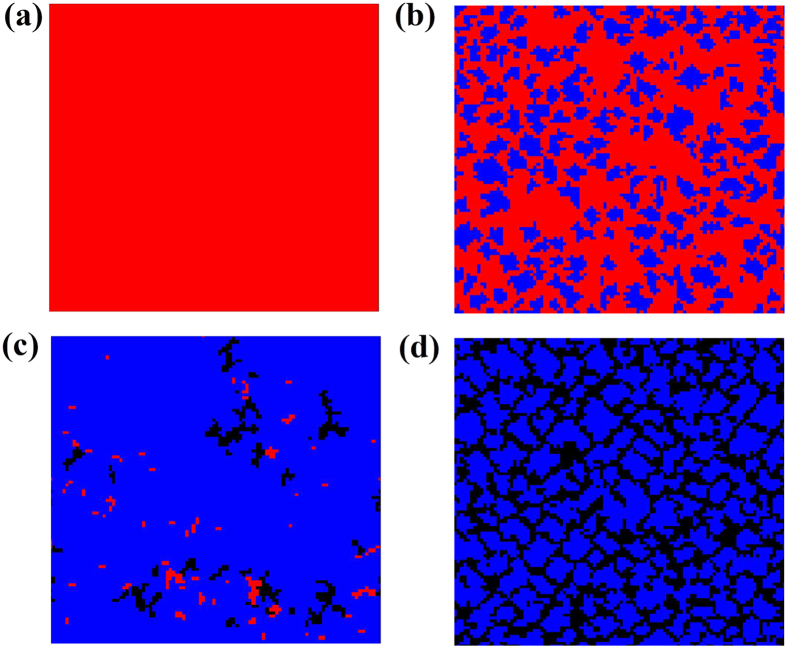
Snapshots of cooperators (black), reward agents (blue) and defectors (red) on a 100 × 100 spatial grid for different values of *γ*. (**a**) The classical version for the PDG. From (**b**) to (**d**), the values of *γ* are 0.1, 0.3, and 0.5. All the results are obtained for *b* = 1.20 and *β* = 0.01.

To explore what are the mechanisms that allow the social reward to favor cooperation, we analyze the typical spatial configurations of the three types of agents for the PDG on a regular square lattice in Fig. 3. The parameter *b* is given by a constant term in the four patterns (*b* = 1.2). In the traditional case, the defectors occupy the whole lattice after 10^5^ MCS (at equilibrium) as shown in Fig. 3(a), which suggests that network reciprocity is not enough to enhance the evolution of cooperation for the applied temptation value. However, when the social reward is introduced into the game, different evolutionary phenomena can be observed.Different from the classic case, a few cooperative agents (reward agents) survive in the equilibrium state as shown in Fig. 3(b) (*γ* = 0.1). It is because the cooperators who get in touch with defectors will firstly become reward agents and form clusters to prevent the invasion of defectors. It is the protection mechanism that allows the cooperation strategy to survive under the temptation value. When the level of reward becomes higher (say *γ* = 0.3 or 0.5), the cooperative agents not only survive but also become the dominant strategy. As illustrated in Fig. 3(c) (*γ* = 0.3), the clusters of reward agents become larger and more compact, which further results in fewer spaces left for the defectors. A few cooperator islands survive in the interior of the giant reward clusters that protect them from the intrusion by defectors. When the value of *γ* is further increased (*γ* = 0.5), for the cooperators, the profits from the interaction with reward agents become larger. Therefore, a few of reward agents turn into the members of cooperators and form a series of compact C + Re clusters, which make the special cooperative agents become more and more powerful. Finally, the cooperators and the reward agents tend to replace the defectors and occupy the whole population, as illustrated in Fig. 3(d) (*γ* = 0.5). These illustrative snapshots demonstrate the fact that when the reward mechanism is involved into the standard PDG, the sustainability of cooperation can be improved via strategic adjustment (say, from the cooperation strategy to the reward strategy) to form the compact hybrid cooperative clusters.

**Figure 4:**
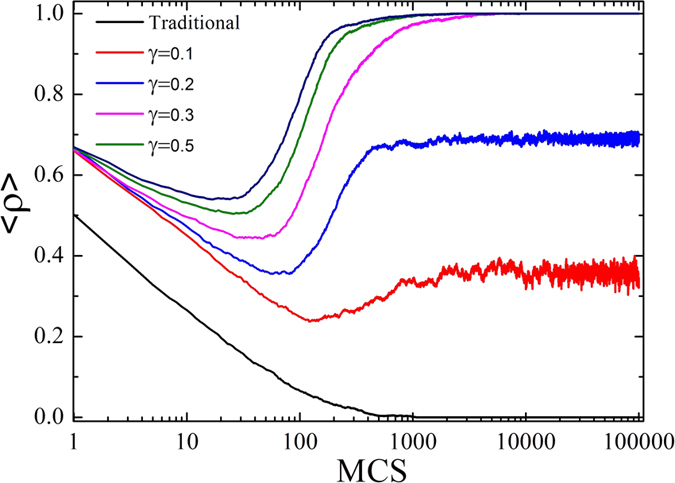
Time course of the fraction of cooperative agents <*ρ*> for the PDG on the square lattice. All the results are obtained for *b* = 1.20, *β* = 0.01. The black curve is corresponding to the traditional case (namely, without reward mechanism), and different colors represent different values of the social reward *γ*.

Note that the reward mechanisms enable the formation of extremely tight compact cooperative clusters. It is significant to elucidate its potential dynamics for the PDG on the square lattice. The temporal traits of the fraction of cooperative agents <*ρ*> for different values of the parameters *γ* are therefore analyzed in Fig. 4. For the traditional version, as shown by the black solid line in Fig. 4, the decrease of the cooperators cannot halt and the cooperative behavior dies out soon. When the reward mechanism is introduced, as the evolution continues, the role of the reward begins to be awaken. It is observed that the exploitation of the defectors is effectively restrained and the spreading of the cooperative behavior is promoted. As shown in the figure, the larger of the *γ* values, the more evident the reversal of cooperation. For a large value of *γ (γ* = 0.3 or 0.5), the initial decay of cooperation is restrained earlier, and finally the cooperation reaches a higher level (more than 99%). Part of the cooperators turn into reward agents and form a few compact cooperative clusters to counterattack against the invasion of the defectors, and the fraction of the cooperative agents begin to rise. The cooperative agents situated at the boundaries of clusters become robust to defector attacks and even induce the weakened defectors to become their compartments, which ultimately causes the undisputed dominance of cooperation. It is worth mentioning that the reward agents, as a defined novel kind of cooperative agents, have the same probability to populate the grid as the traditional cooperators and defectors in the initial conditions. That is why the fraction of cooperative agents in the initial condition under the newly introduced mechanism is higher than the fraction of cooperators in the standard game model.

**Figure 5:**
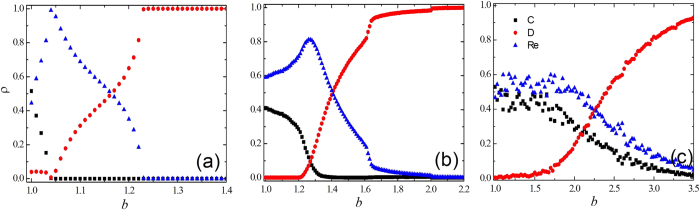
The fraction of cooperators, reward agents and defectors <*ρ*> dependent on the temptation to defect *b* for three types of topologies. From (**a**) to (**c**) the networks are square lattice, ER and SF networks, respectively. All the results are obtained for *γ* = 0.1 and *β* = 0.01.

Then it will become of particular interest to confirm the above analysis of the evolution processes. In order to provide accurate answers, we investigate in Fig. 5 the fractions of the three independent strategies including cooperation strategy, defection strategy and reward strategy in the three network topologies. The numerical simulation results of evolutionary PDG on the square lattice, the ER and SF networks are presented from Fig. 5(a) to Fig. 5(c), respectively. The values of *γ* in the three graphs are fixed to be 0.1. The change trends of the three strategies on the regular network (Fig. 5(a)) and the ER network (Fig. 5(b)) are extremely similar. When the *b* value is low, there are few defectors populating on the networks, and cooperative agents occupy most of the network nodes. With the increase of *b*, cooperators gradually convert to reward agents to resist the aggression of the defectors till the number of reward agents reaches the maximum value (all the cooperators turn into reward agents). The value of *b* corresponding to the maximum number of reward agents is 1.04 for the square lattice and 1.26 for the ER network, which also indicates that the evolution of cooperation on the ER network is superior to the evolution on the square lattice. Once all the cooperators become reward agents, these strategists have no other way to stop the spreading of defectors and the invasion of defectors become overwhelming. Essentially, because the interaction between reward agents reports fewer benefits than those between a defector and a reward agent, a small increase in *b* induces a transition for the agents from reward strategy to defection strategy, which finally produces the breakdown of the reward agent clusters into small clusters till all the reward agents die out. Ultimately, the defectors occupy the entire network.

The results for the SF network are radically different from the evolution of cooperative behavior on the ER and square lattice networks, as presented in Fig. 5(c). Both the reward agents and cooperators are monotonically decreasing as *b* becomes large. Meanwhile, the number of defectors is increasing monotonically, which implies that the mechanism promoting the evolution of cooperation for SF network may be different from the two previous discussed networks. From the change trends of the fractions of the three strategists, we can speculate that when the value of *b* is low, the system is composed of numerous islands of cooperators surrounded by reward agents. When the temptation to defect *b* gradually becomes larger, the profits from the interaction between reward agents are fewer than those from the interaction between a defector and a cooperator. Therefore, the reward agents who are situated on the vertices among the cooperators and the defectors will firstly convert to defectors. Those cooperators who are protected by reward agents become defectors as well, which induces the gradual growth of the number of defection clusters, as Fig. 5(c) shows.

**Figure 6:**
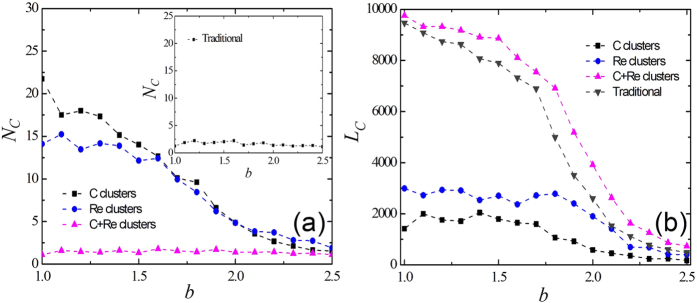
Number of clusters of pure cooperative agents *N*_*C*_ and number of the largest cluster of cooperative individuals (*L*_*C*_) dependent on *b* for SF topologies. The value of *γ* is 0.1 and *β* = 0.01.

The local distributions of the cooperators provide us with important clues to analyze the system at the microscopic scale. Therefore, a pure strategist who keeps its strategy unchanged in all generations after a transient period is defined. Generally speaking, we pay more attention to cooperative behaviors, and we define three kinds of pure strategists: pure cooperators, pure reward agents, and pure cooperators and reward agents (defined as the agents that alternatively spend some time as a cooperator or as a reward agent). To further confirm the above analysis of the evolutionary mechanism for the PDG on the SF topologies, we pay attention to the three types of clusters of pure strategists: clusters formed by pure cooperators (C clusters), clusters formed by pure reward agents (Re clusters) and the ones formed by pure cooperators and reward agents together (C + Re clusters). In particular, the C + Re clusters may contain several C clusters or Re clusters. The evolution of the number of pure cooperative clusters (*N*_*C*_) and the number of cooperative agents (*L*_*C*_) in the corresponding largest cluster *N*_*C*_ as a function of the temptation to defect *b* are presented in Fig. 6(a) and Fig. 6(b). In the standard PDG on SF graphs, hubs are usually occupied by the cooperators and a giant cluster of cooperators starts to grow around them until the entire network forms a complete cluster. Increasing *b* produces a reduction in the size of the cooperation cluster that doesn’t collapse until a higher *b* is reached. As inferred before, the number of cooperation and reward clusters monotonically decreases while only one cooperation and reward cluster is presented in the system until it dies out for higher b values as shown in Fig. 6(a). This is in agreement with the standard formulation of the PDG on SF topologies. Furthermore, it is evidently demonstrate in Fig. 6 that the evolution of cooperation is improved by social reward through comparison between the traditional results and the simulation results under the reward mechanism. The results imply that in the presence of social reward, the heterogeneity of the topologies strongly affects the structure and evolution of cooperation.

**Figure 7:**
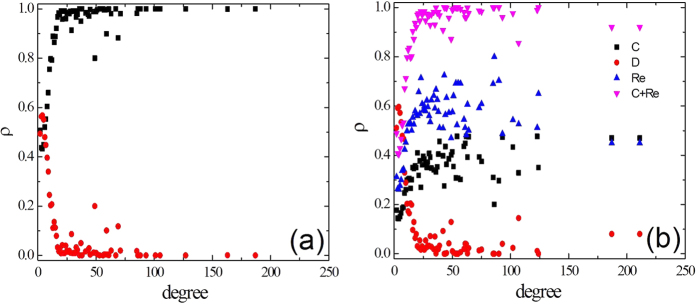
Distribution of strategies on SF topologies. The data represent the ratios of cooperators, defectors and reward agents as a function of the nodes’ degrees at equilibrium. (**a**) The traditional version of the PDG (*b* = 2.25). (**b**) Results for our model with social reward for the PDG (*β* = 0.01, *γ* = 0.1 and *b* = 2.4).

Finally, we have also explored how the three strategists are distributed by degree classes. The distributions of cooperators, defectors and reward agents at equilibrium for degree classes on the SF networks are demonstrated in Fig. 7(a) and Fig. 7(b), which represent for the traditional PDG (*b* = 2.25) and the evolutionary PDG (*b* = 2.4) in the newly introduced mechanism (*γ* = 0.1), respectively. The selection of parameter *b* is made to let the cooperation level equal in the two models, where the fraction of cooperation approximately equal to 0.5. The ratios of cooperators (C) and the mix of cooperators and reward agents (C + Re) as a function of the nodes’ degrees in Fig. 7(a) and in Fig. 7(b) imply that pure cooperators in the traditional model are mainly replaced by the mix of ‘C’ and ‘Re’ players, and there are only very few new nodes with different degrees form. As the figure shows, the cooperators and reward agents tend to dominate in moderate and highly connected nodes, which is also more conducive to the formation of large clusters to resist the invasion of defectors. That is why the SF network has more advantages in promoting the evolution of cooperation compared with the other two network topologies (the regular lattice and ER networks) as illustrated in Fig. 1 and Fig. 2.

## Discussion

The reward scenario is extremely common in realistic world, ranging from biological systems to economic as well as social systems. In this context, we have explored the impact of social reward on the evolution of cooperation in prisoners’ dilemma game and snowdrift game. Numerical simulations show that when the social reward is introduced, the cooperation strategy is greatly improved in both the PDG and the SDG.

The analysis of the system at the microscopic level for the PDG helps us to understand the mechanisms that favor the evolution of cooperation. As described in many classical works^55^,^56^,^57^,^58^, the formation of cooperator clusters is an essential way to resist the invasion of defectors. In the three-strategy model, the defined cooperative players (reward agents) make the individuals more inclined to choose cooperation when b is small, which might be assumed to be conducive to the formation of cooperative clusters. With the increment of the temptation to defect, the cooperators firstly turn into reward agents, and then the giant cluster of reward agents break down into several ones until all the reward agents die out. Finally, defection becomes the dominant strategy. While in heterogenous graphs, cooperation is driven by nodes that can be both cooperators or reward agents. When b continues to increase, benefit from cooperative strategy is less than that from defection strategy. Therefore, the cooperative agents who are situated on the vertices among cooperators and defectors will firstly convert to defectors. By erosion of the single cluster of cooperators, the defectors gradually occupy the whole network.

In reality, individual behavior can be guided in a certain direction by social reward just as that in the article. Our results suggest that the reward strategy has a beneficial impact on the evolution of cooperative behavior, which might be particularly essential for animal and human societies.

## Methods

As mentioned in the *Introduction* section, the three typical social dilemmas involving pairwise interactions include the prisoner’s dilemma game, the snowdrift game and the stag-hunt game. Here, we focus on the prisoner’s dilemma game (PDG) and the snowdrift game (SDG). We introduce social reward in the PDG and the SDG models on three network structures. In this work, the reward is presented as an additional third strategy corresponding to a novel kind of cooperative agents defined as reward agents. When playing with a cooperator, the reward agent will reward the cooperator *γ* at a small cost *β*, while in interaction with a defector, the reward agent acts as a cooperator. Therefore, the reward agents are defined as particular cooperators. However, note that they can exist independently, which is at variance with the second-stage behavior^51^. It is found from the simulation results (see Supplementary Fig. S1 and Fig. S2) that only when the value of *β* is smaller than *γ*, will the reward mechanism promote the evolution of cooperation. When *γ* is fixed, the smaller the value of *β*, the more obvious the promoting effect. In the model, we fix *β* to be 0.01 and change *γ* from 0.1 to 0.9 assuring that an obvious promoting effect can be observed. Based on previous seminal works, we consider the evolutionary PDG that is characterized with the temptation to defect *T* = *b* (the highest payoff received by a defector when playing against a cooperator), reward for mutual cooperation *R* = 1, and both the punishment for mutual defection *P* as well as the suckers payoff *S* (the lowest payoff received by a cooperator if playing against a defector) equaling 0. It should be pointed out that although we choose a simple and weak version (namely, *S* = 0), the conclusions are robust and can be observed in the full parameterized space^59^. For the SDG, we choose a similar scheme with *T* = 1 + *r, R* = 1, *S* = 1 − *r* and *P* = 0, where 0 ≤ *r* ≤ 1 represents the cost-to-benefit ratio (satisfying the ranking *T* > *R* > *S* > *P*). The interactions between the agents and the relative payoffs are presented in Table 1.

The evolutionary games will be conducted in regular square lattices with periodic boundary conditions, Erdös-Rényi (ER) graph, and Barabási-Albert scale-free (SF) network with the same size (*N* = 10^4^ nodes) and the same average degree, i.e., <*k*> = 4. Initially, the three strategies of cooperation (C), defection (D), and reward (Re) are randomly distributed among the individuals with an equal probability. Then, at each Monte Carlo time step, each node *i* in the network gets a payoff *P*_*i*_ by adding all the payoffs obtained from playing with all its neighbors. Next, all the agents synchronously upstate their strategies by comparing the respective payoffs with their randomly selected neighbors, say *j*, and if *P*_*i*_ > *P*_*j*_, player *i* will keep its strategy unchanged in the next step. On the contrary, if *P*_*i*_ < *P*_*j*_, player *i* will adopt the strategy of player *j* with a probability proportional to the payoff difference:


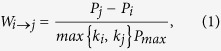


where *k*_*i*_ and *k*_*j*_ represent the degrees of players *i* and *j* respectively, and *P*_*max*_ stands for the maximum possible payoff difference between the two agents. For the classical version (without reward mechanism), it is obvious that *P*_*max*_ equals to *b* (1 + *r*) for the PDG (SDG). However, under the newly introduced mechanism, the value of *P*_*max*_ depends on the size relationship between *b* (1 + *r*) and 1 + *γ*. From equation (1), it is worth noting that it is possible for one individual to change its strategy into another strategy if the payoff of the chosen neighbor is higher than its. For instance, a cooperator can become a reward agent or a defector, but it is not a consequence of a sort of second-stage behavior in this evolutionary dynamics.

The simulations results are acquired by averaging over the last 10^4^ Monte Carlo time steps of the total 10^5^. To double-check, we further analyze the size of the fluctuations in <*ρ*>. If the size is smaller than 10^−2^, we assume that the stationary state has been reached; otherwise we wait for another 10^4^ time-steps and redo the check. The system reaches the stationary state in all the simulations and no additional time-steps are needed.

## Additional Information

**How to cite this article**: Wu, Y. *et al*. Impact of Social Reward on the Evolution of the Cooperation Behavior in Complex Networks. *Sci. Rep.* **7**, 41076; doi: 10.1038/srep41076 (2017).

**Publisher's note:** Springer Nature remains neutral with regard to jurisdictional claims in published maps and institutional affiliations.

## Supplementary Material

Supplementary Information

## Figures and Tables

**Table 1:** Payoff matrix of the studied evolutionary game.

	C	D	Re
C	R	S	R + *γ*
D	T	P	T
Re	R − *β*	S	R + *γ* − *β*

The three strategies are cooperation (C), defection (D), and reward (Re), respectively. Here, *γ* stands for the reward applied to cooperators and *β* is the cost of reward.
