# Supplementary material for: Impact of Social Reward on the Evolution of the Cooperation Behavior in Complex Networks
Source: Sci Rep. 2017 Jan 23;7:41076. doi: 10.1038/srep41076 (PMC5253654; doi:10.1038/srep41076)
Supplement: Supplementary Information [file srep41076-s1.pdf]

Impact of Social Reward on the Evolution of the Cooperation Behavior in Complex Networks

YU'E WU,[1] SHUHUA CHANG*,[1,a)] ZHIPENG ZHANG,[1] and ZHENGHONG DENG[2]

[1)] *Coordinated Innovation Center for Computable Modeling in Management Science, Tianjin University of Finance and Economics, Tianjin 300222, China*

[2)] *School of Automation, Northwestern Polytechnical University, Xi'an 710072, China*

(Dated: 6 December 2016)

[a)] shuhua55@126.com

FRACTION OF COOPERATIVE AGENTS IN THE PRISONER'S DILEMMA

Here, we fix γ at 0.3 and change β from 0.0(traditional version) to 0.4. The fraction of cooperative agents (cooperators and reward agents) $<\rho>$ as a function of b for different values of β for the prisoner's dilemma on square lattice is shown in Fig. S1. It is found that only when the value of β is smaller than γ, will the reward mechanism promote the evolution of cooperation. However, it is observed that this promoting effect starts with a certain b value rather than appears initially. Through analysis, we find that there are only cooperators and defectors in the system at equilibrium before the b value, which makes the evolution of cooperation in the three-strategy model coincide with the case in the traditional version. In addition, only when b is greater than this value, will the reward agents appear. This phenomenon may be related to the benefit relationship between agents when they interact with each other for the applied b-value. Although this is a problem worthy of further discussions, this does not affect our conclusions.

FRACTION OF COOPERATIVE AGENTS IN THE SNOWDRIFT GAME

For the snowdrift game on the square lattice, the results are similar with the prisoner's dilemma. The parameters are the same too.

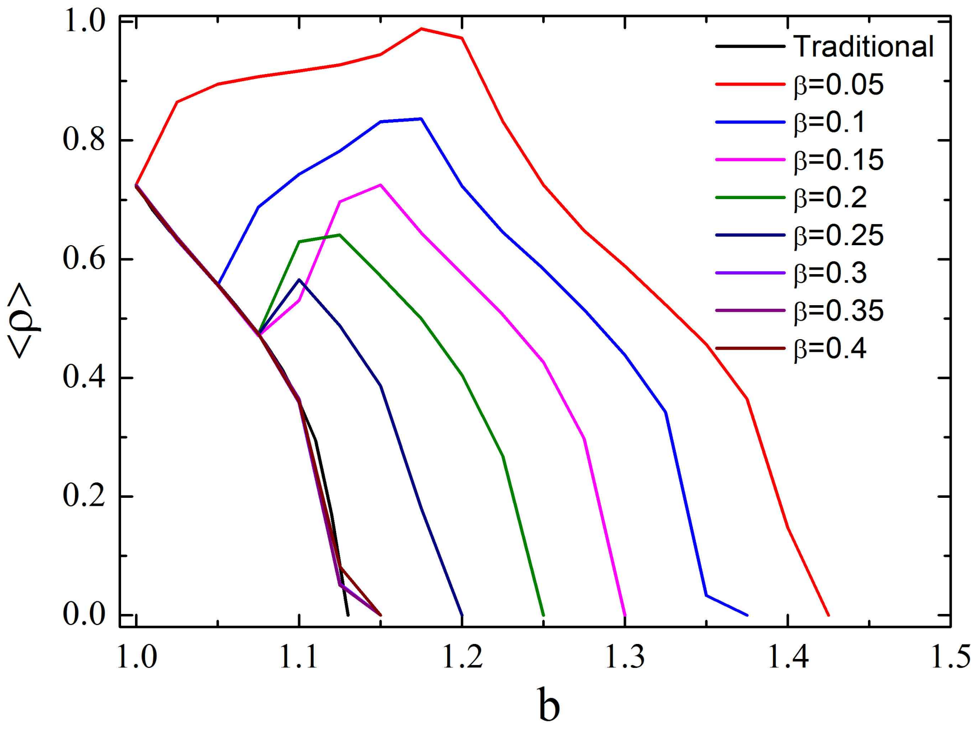

Fig. S1. **Fraction of cooperative agents (cooperators and reward agents) $<\rho>$ in the prisoner's dilemma as a function of b for different values of β.** The results are obtained for γ=0.3 and N=10^4 nodes.

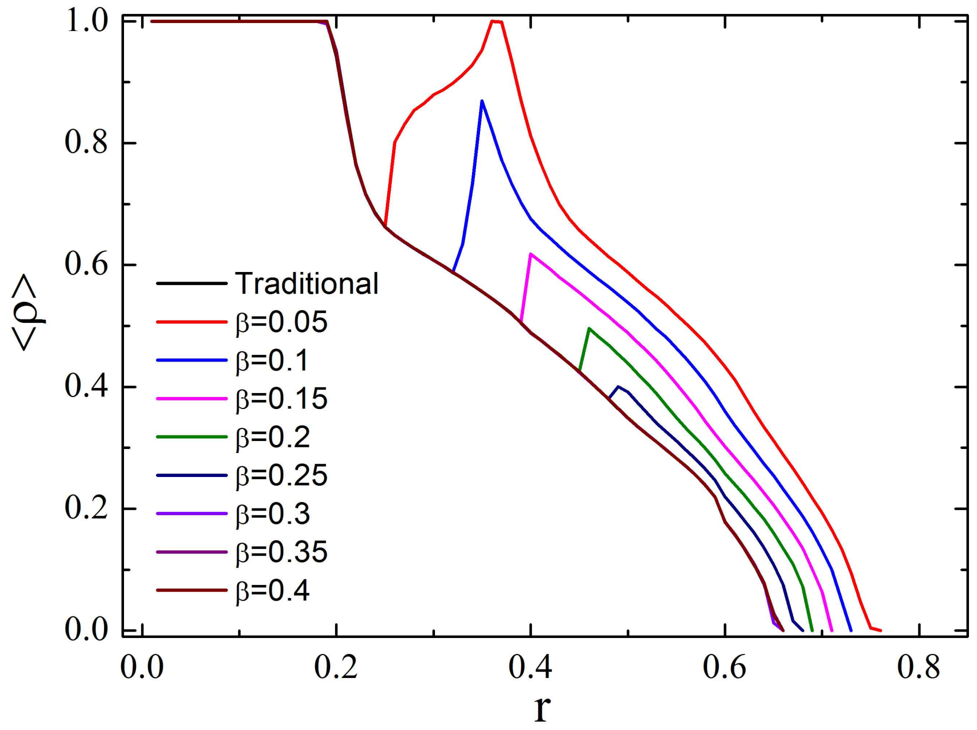

Fig. S2. **Fraction of cooperative agents (cooperators and reward agents) $<\rho>$ in the snowdrift game as a function of the cost-to-benefit ratio r for different values of β.** Other parameters are the same as in Fig. S1.
